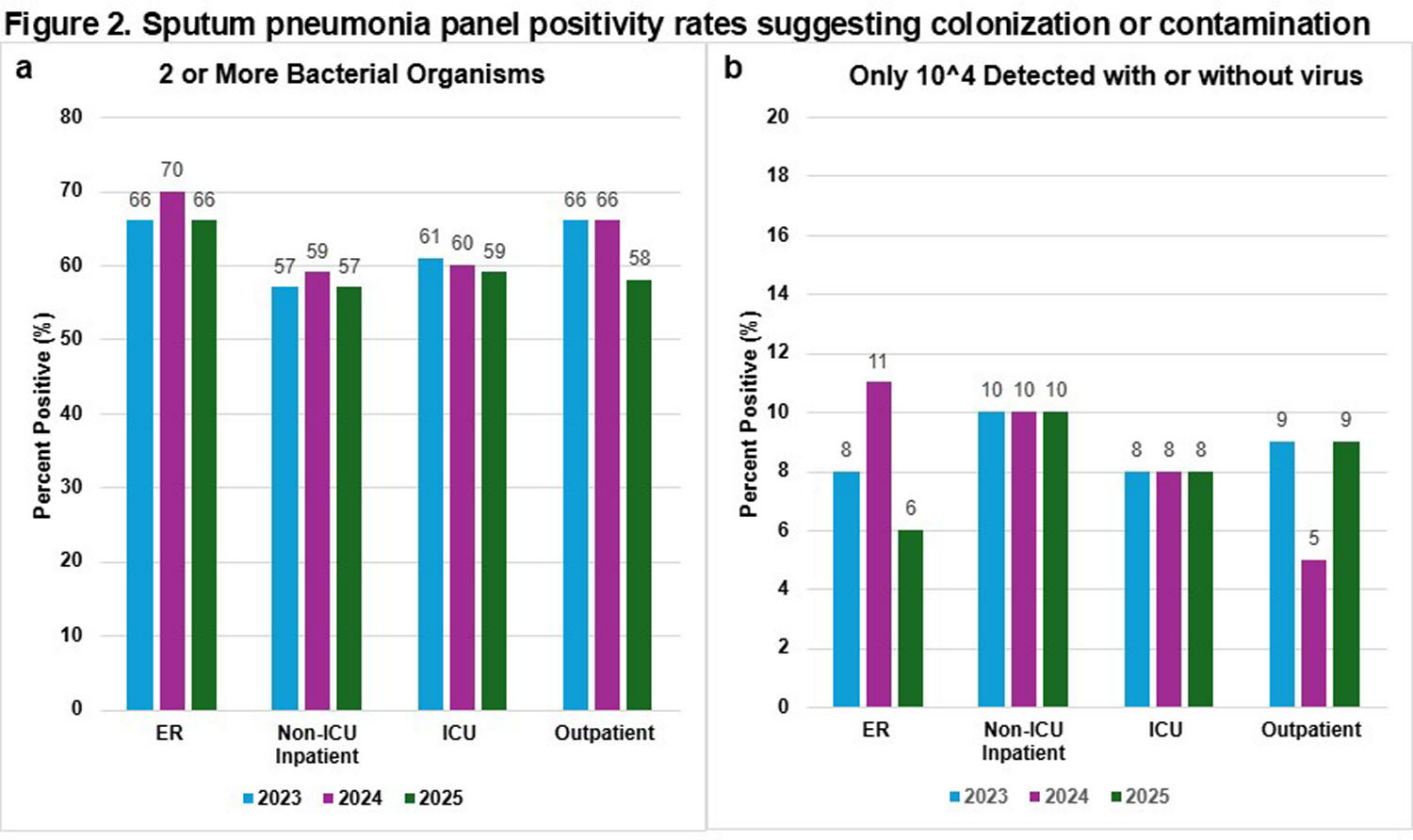# 209 Evaluation of AI Chatbot Responses to Simulated Caregiver Questions About Asymptomatic Bacteriuria

**DOI:** 10.1017/ash.2026.10594

**Published:** 2026-06-23

**Authors:** Madeline DiLorenzo, Kassandra Marsh, Guiqing Wang, Caitlin Otto, Ioannis Zacharioudakis, Dana Mazo

**Affiliations:** 1 NYU Langone Health

## Abstract

**Background:** Multiplex syndromic polymerase chain reaction (PCR) panels can rapidly identify pathogens in patients with diagnosis of pneumonia, but they may also detect colonizing bacteria, which can lead to diagnostic uncertainty. Their utility in sputum samples remains unclear, as most evidence comes from higher-quality bronchoscopy specimens. To ensure these tests are used in scenarios with the greatest clinical impact, such as when results can be returned quickly and guide treatment, we developed a clinical decision support tool. The tool restricts ordering of multiplex pneumonia PCR panel on sputum samples to patients with severe illness or immunocompromise. **Methods:** The study was conducted at a New York City hospital system. Beginning in December 2024, the pneumonia PCR panel decision support tool was implemented and restricted ordering on sputum samples to patients who were admitted to the intensive care unit (ICU), prescribed vasopressors, required at least high flow nasal cannula for supplemental oxygen, had solid organ or hematopoietic transplant, received Gram negative coverage with meropenem or broader for 48 hours without improvement, and/or infectious diseases or pulmonary attending consult recommendation. We analyzed the total number of sputum pneumonia PCR panels and associated positivity rates prior to and after implementation of the decision support tool during quarters 1 through 3 of 2023, 2024 and 2025. **Results:** After intervention implementation, the overall number of sputum pneumonia PCR panels ordered decreased by 34%. At $200 per PCR panel, this represents a cost savings of at least $208,000. The greatest change was on non-ICU inpatients, where the total decreased from 1267 in 2024 to 504 in 2025 (60%, Figure 1). The emergency department had a decrease from 266 in 2024 to 125 in 2025 (53%). There was minimal change in PCR panel usage in the ICU and outpatient, which are areas not targeted by the intervention. The positivity rate was consistent over the three years (70-75%). Most sputum PCRs detected multiple bacterial organisms (57-70%, Figure 2a) and 5-11% of samples detected bacteria at a quantity of only 10^4, with or without viral co-infection (Figure 2b). After reviewing post-implementation quarter 1 2025 data, broad Gram-negative coverage was removed as a testing criterion since it was selected frequently (32%) but not accurately (10% accuracy). **Conclusion:** Implementation of a clinical decision support tool in Epic led to more appropriate diagnostic stewardship of the multiplex pneumonia PCR, improving care and reducing costs. Future interventions will target improving stewardship in the ICU.

**Figure:**
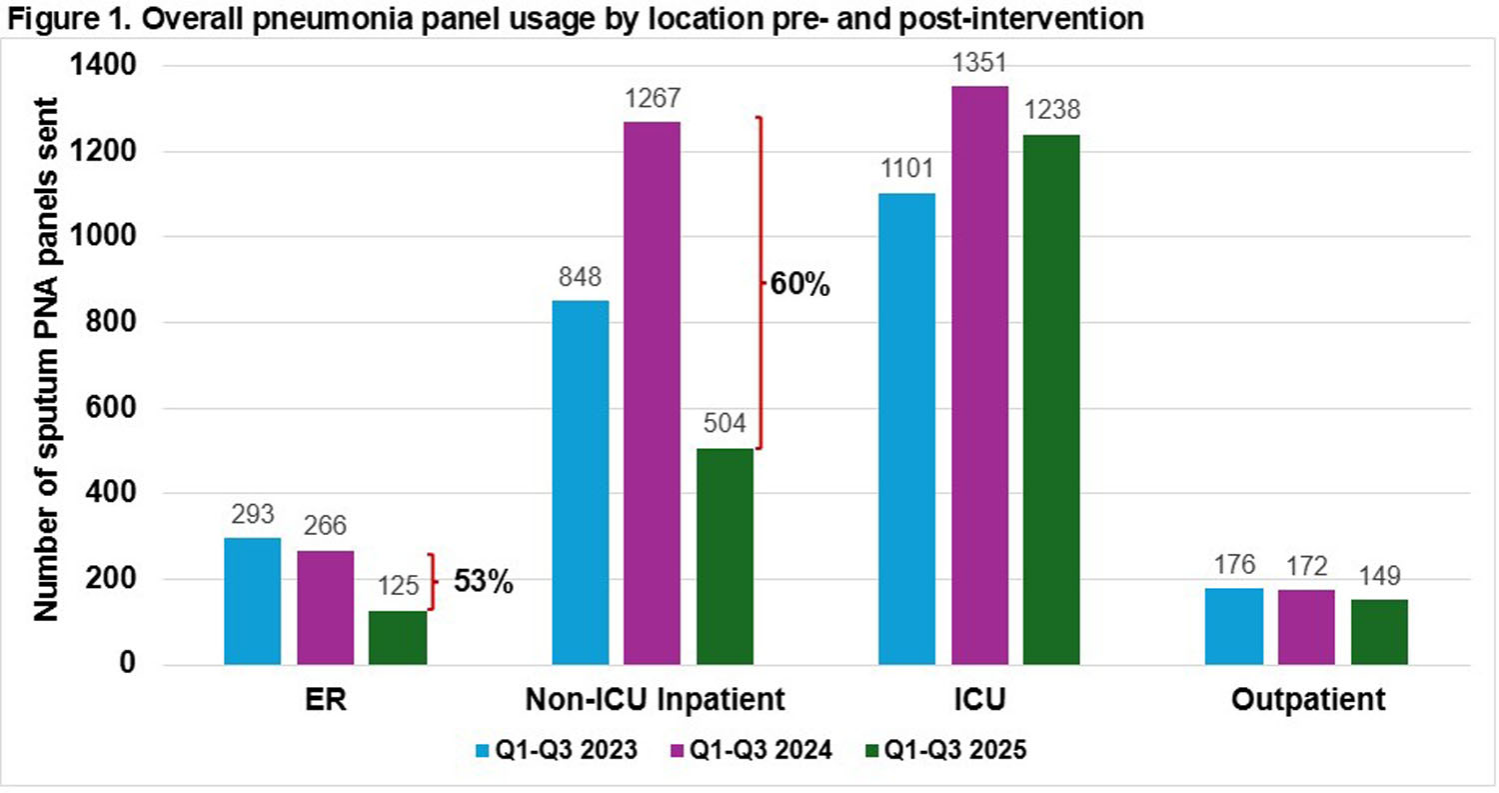

**Figure:**